# Bullying and Cyberbullying in Adolescents from Disadvantaged Areas: Validation of Questionnaires; Prevalence Rates; and Relationship to Self-Esteem, Empathy and Social Skills

**DOI:** 10.3390/ijerph17176199

**Published:** 2020-08-26

**Authors:** Jacinto Martínez, Antonio J. Rodríguez-Hidalgo, Izabela Zych

**Affiliations:** 1 Department of Psychology, University of Cordoba, Avda. San Alberto Magno S/N, 14071 Córdoba, Spain; jacintomasa@gmail.com (J.M.); izych@uco.es (I.Z.); 2 Department of Psychology, Cátedra de Cooperación al Desarrollo, University of Cordoba, Avda. San Alberto Magno S/N, 14071 Córdoba, Spain

**Keywords:** bullying, cyberbullying, self-esteem, empathy, social skills

## Abstract

Although bullying and cyberbullying have been widely studied in diverse geographical areas, the number of studies in isolated regions, located in rainforests such as the Peruvian Amazonia, is low. Most research has been conducted in wealthy, Western countries, although disadvantaged areas are usually the most affected by various problems. Thus, the aims of this study were to validate bullying and cyberbullying measurement instruments among adolescents in the Peruvian Amazonia, to determine the prevalence rates of bullying and cyberbullying among this population, and to examine how bullying and cyberbullying relate to self-esteem, empathy, and social skills. The sample included 607 students from the region of Loreto (Peruvian Amazonia) who completed self-report questionnaires. Both questionnaires used in the sample were found to have good psychometric properties. Results showed that bullying and cyberbullying are prevalent among teenagers in the Amazonia. Low self-esteem and high affective empathy predicted bullying victimization. Being a bully was related to high assertiveness. Being a bully-victim was related to low self-esteem and low assertiveness. Cybervictims showed higher cognitive empathy. Cyberbullies showed higher affective empathy in comparison to uninvolved adolescents. Having low self-esteem and higher affective empathy were related to being a cyberbully/victim. This study provides a validated questionnaire that can be used for research and practice in the Amazonia. Based on the current results, tailored anti-bullying and anti-cyberbullying interventions with components focused on self-esteem, empathy, and social skills should be implemented in Peruvian secondary schools.

## 1. Introduction

Bullying and cyberbullying have been studied for over four decades. However, a systematic review showed that, up to the year 2015, more than 75% of bullying studies had been conducted in Europe and the United States [1]. Although describing, understanding, and reducing violence—including bullying—is necessary worldwide, it is especially important in disadvantaged geographical regions [2,3]. Hence, the current study focuses on bullying and cyberbullying in the Peruvian Amazonia, an area which, to our knowledge, has not yet been studied in relation to bullying and cyberbullying.

Bullying is defined as repeated, long-term behavior involving certain students intentionally acting aggressively towards peers who cannot defend themselves easily [4]. Bullying is based on a dominance–submission relationship [5] that allows the perpetrator to avoid punishment and makes the victim more vulnerable, and frequently unable to escape from this situation [6]. Bullying is a complex psychosocial group behavior that includes different roles [7]. Victims are the students who are repeatedly abused, and bullies are the students who perpetrate aggressive behaviors towards their peers. Bully–victims are the students who are bullied and bully others at the same time, and there are also bystanders who reinforce the perpetrator’s behavior.

The growth of technology brings many opportunities, but it also provides a context for a new form of bullying, called cyberbullying. Cyberbullying shares many characteristics with face-to-face bullying, such as being intentionally harmful, being repetitive, and involving a power imbalance. However, cyberbullying is perpetrated via electronic devices, can take place 24 h a day, 7 days a week, allows perpetrators to remain anonymous, and is more likely to have a bigger audience [8,9]. Cyberbullying does not require the perpetrator to repeat their violent actions, as one or two cyberaggressions can remain online for long periods of time [10], thus lengthening the victim’s suffering [11].

Bullying and cyberbullying have serious short-, medium-, and long-term consequences [1]. Moreover, they are related to other antisocial behaviors, according to both longitudinal and cross-sectional studies [12]. Reducing violence and other antisocial behaviors in minors has become one of the top scientific, political, and practical priorities in social sciences. However, only 10% of studies with a focus on different types of violence have been conducted in low- and middle-income countries, even though 85% of violent deaths occur in low- and middle-income countries [13]. The number of studies on bullying and cyberbullying in disadvantaged areas is also low [14] and, at the same time, understanding the subject is the key to reducing violence globally. For these reasons, it is necessary to validate instruments, to examine bullying and cyberbullying in disadvantaged regions, and to uncover possible risk and protective factors.

A review on bullying and cyberbullying research in Latin America [15] showed that there are still many gaps in knowledge in that geographical area. Regarding cyberbullying, the number of studies conducted in Latin America is still low, and a small percentage of them are published in high-impact journals. Regarding both bullying and cyberbullying, several high-impact articles about the validation of questionnaires in countries such as Colombia [16,17] and Ecuador [3] have been recently published. Latin America, however, is a large, socioeconomically diverse, and culturally diverse geographical area, and it is necessary to validate instruments in its different countries and regions.

### 1.1. Prevalence Rates of Bullying and Cyberbullying: Research in Disadvantaged Areas

A global study with 104,614 students from 19 countries showed that 34% of the respondents reported bullying victimization [18]. Other international studies reported that approximately one out of three minors was involved in bullying and one out of five was involved in cyberbullying [1,19]. Another review of cyberbullying research has shown that 56.9% of survey respondents had been victimized in the last 6 months and 65% had been victimized at least once during their lifetime [20]. A cross-European study found overall cyberperpetration prevalence rates of 17.7% in males and 10.9% in females, and overall cybervictimization rates of 16.5% in males and 16.4% in females [21].

Research has shown that there is more violence in schools in Latin America than in Europe or the United States [22]. A review that analyzed prevalence rates of bullying across 234 Latin American articles found the average prevalence rate of bullying was 29.31% and the average prevalence rate of cyberbullying was between 2.5% and 42.5% [15]. A Latin American study conducted by The United Nations Children’s Fund (UNICEF) found that between 50% and 70% of students were involved in bullying [23]. With respect to cyberbullying, a study conducted in 16 Latin American countries reported prevalence rates between 13% and 63% [24]. A more recent study by Cabra and Marciales [25] concluded that there is still little information about bullying and cyberbullying in Latin America, and, therefore, no conclusion regarding overall prevalence rates can be drawn. Moreover, most studies in Latin America included students from Brazil, Colombia, Chile, and Mexico [15]. Therefore, it is still necessary to conduct research on bullying and cyberbullying in different Latin American regions, especially in disadvantaged areas.

Peruvian Amazonia is one of the regions where research on bullying and cyberbullying is still needed. The Peruvian Ministry of Health [26] reported that 38% of Peruvian students had been physically attacked in the previous year and that 47.5% had been victims of bullying in the previous month. According to the Peruvian anti-bullying platform SiseVe (https://siseve.pe), from 2013 to 2018, a total of 22,126 cases of school violence were reported, with 55% of them considered bullying [27]. The National Peruvian Survey on Social Relationships [28], conducted in 2015, showed that 73.5% of children and 73.8% of adolescents had suffered some type of violence during their school years. Another study, with teenagers between 12 and 17 years of age, revealed that 47% of them had been victims of violence perpetrated by peers in the past year [29]. As for cyberbullying in Peru, victimization prevalence rates of 31.3% were found [30], while other studies showed a 24.6% prevalence rate [31]. Another study, with almost one thousand Peruvian students between 9 and 11 years of age, found that between 0.7% and 3.2% of them had occasionally witnessed cyberbullying [32].

Thus, several studies have revealed that bullying and cyberbullying do exist in Peru, although prevalence rates vary across studies. Even though some preliminary results about bullying and cyberbullying in Peru have been published, to our knowledge, there are still no validated questionnaires to measure bullying and cyberbullying in Peru. Validated questionnaires are indispensable for collecting data with scientific rigor. Moreover, a review of scientific literature shows that there are no projects with samples coming from particularly disadvantaged areas, such as the Peruvian Amazonia.

### 1.2. Bullying and Cyberbullying: Relation to Self-Esteem, Empathy, and Social Skills

Several studies have revealed that self-esteem is related to bullying and cyberbullying. A recent meta-analysis found that peer violence predicted low self-esteem, and that low self-esteem predicted more peer violence [33]. A literature review performed by Kowalski [34] concluded that low self-esteem is a risk factor for cybervictimization, and that high self-esteem can be a protective factor against it. The relation between low self-esteem and victimization might be explained by the fact that perpetrators look for victims who are less capable of defending themselves [35].

Other studies have shown that low self-esteem is related to aggression [36]. A systematic review of meta-analyses on the protective factors against bullying and cyberbullying discovered that high self-esteem protects against involvement in different bullying and cyberbullying roles [37]. Another meta-analysis [38] found that high self-esteem was related to decreased bullying victimization and perpetration. Meta-analyses conducted by Chen et al. and Kowalski et al. [39,40] found relations between high self-esteem and decreased victimization, and perpetration of cyberbullying. Other studies found that low self-esteem was related to self-victimization and ethnic-cultural victimization [41].

Scientific results regarding the relations between empathy, bullying, and cyberbullying are inconclusive. In general, high empathy has been described as a protective factor against bullying [37,42], while low empathy has been described as a risk factor for bullying [37,43,44,45]. However, some studies have found low levels of empathy in bullies and high levels of empathy in victims [37,46]. A meta-analysis found low empathy in cyberbullies and high affective empathy in cybervictims [37]. High affective empathy in cybervictims was also found in previous research [47]. There are also studies that did not find a significant relation between empathy, bullying, and cyberbullying [37,40].

These inconsistent results have been explained in several different ways. On the one hand, it has been suggested that people with high levels of empathy can better understand the victim’s feelings, which prevents them from being abusive [48]. Given that high affective empathy has been related to victimization [49], it is possible that victims are more sensitive because they suffered more than the uninvolved students. Regarding bullies, it is also possible that they do understand and even share others’ feelings but continue to harm victims as their character may be Machiavellian and manipulative [50].

In addition to self-esteem and empathy, social skills have also been studied in relation to bullying and cyberbullying. Social skills are described as an essential tool for proper adolescent development, social adjustment, and social relationships [51]. Some studies have found a relationship between low social skills and involvement in bullying [52,53,54,55]. Additionally, different social and emotional competencies were found to be protective against bullying and cyberbullying [37]. Various meta-analyses have shown that social competencies, together with conflict resolution skills, relate to lower victimization [40,56], and other studies have found that social skills are protective against the possibility of becoming bullies and bully-victims [56]. Scientific literature has shown that an increase in different social skills, particularly conflict resolution, is related to a decrease in bullying [57]. More assertiveness is related to less aggression and to defending the victims of bullying [58]. It has also been found that low social abilities predict more bullying [59] and peer victimization [60].

Reviews of the scientific literature on the relations between self-esteem, empathy, social skills, and violence (including bullying and cyberbullying) show that there is an emerging research line focused on bullying and cyberbullying in disadvantaged areas. A study in Ecuador found that low self-esteem, as well as high levels of affective and cognitive empathy, predicted high levels of bullying victimization [3]. However, victimization was not predicted by social skills. They also found that bullying perpetration was predicted by low self-esteem and low cognitive empathy. Social skills did not predict bullying. Nevertheless, it is necessary to advance knowledge in the field over different disadvantaged geographical areas, and to design effective intervention and prevention programs.

### 1.3. Current Study

Reviews of the scientific literature found that bullying and cyberbullying are present and prevalent in different countries. Several studies showed relations between bullying, cyberbullying, empathy, self-esteem, and social skills. However, it is remarkable that the number of studies in Latin America is lower than in Europe or in the United States. Moreover, bullying and cyberbullying have not yet been studied in many disadvantaged and isolated regions, such as the rainforest region. Although some studies have shown high prevalence rates of bullying and cyberbullying in disadvantaged areas, there are still no validated instruments to measure bullying and cyberbullying in most of these regions.

Empathy, social skills, and self-esteem are studied as possible protective factors against bullying and cyberbullying around the world. Thus, it is important to discover if they can also protect adolescents in disadvantaged areas from bullying. This is particularly important, because most violent acts are committed in regions where the number of studies about violence is extremely low.

Thus, this study aims to: 1. Validate the European Bullying Intervention Project Questionnaire and the European Cyberbullying Intervention Project Questionnaire [61] in Peruvian Amazonia; 2. Describe the prevalence rates of different bullying and cyberbullying roles in Peruvian Amazonia; and 3. Discover direct and unique relationships between self-esteem, empathy, and social skills, and the different bullying and cyberbullying roles. It is expected that the questionnaires have good psychometric properties; we also expected to find relatively high prevalence rates of bullying and cyberbullying; and a relationship between high self-esteem, empathy, and social skills, and low involvement in bullying and cyberbullying.

## 2. Method

### 2.1. Participants

The sample of this study consisted of 607 students from four different state schools in the city of Iquitos, region of Loreto (Peruvian Amazonia). Participants were between 12 and 19 years old (Mean age = 14.5, SD = 1.69), and were enrolled in one of the five grades of Compulsory Secondary Education in Peru.

The sample consisted of 51.7% males and 48.3% females. Out of these students, 22.1% were enrolled in Grade 1, 22.1% in Grade 2, 17.3% in Grade 3, 19.9% in Grade 4, and 18.6% in Grade 5. The sample was widely diverse; 73.5% of the students were from the majority ethnic-cultural group (mixed-ethnicity), 20.4% were Caucasian, 5.9% were Native American, and 0.2% were from other ethnic-cultural groups.

### 2.2. Instruments

The first part of the questionnaire consisted of questions related to the different sociodemographic variables such as school, gender, age, grade, and ethnic-cultural group. The rest of the variables were measured with the following questionnaires.

Bullying was measured with the Spanish version of the European Bullying Intervention Project Questionnaire (EBIP-Q) [61,62]. The questionnaire has 14 items, seven of which refer to victimization (e.g., ‘A classmate has punched me, kicked me or pushed me’) and seven of which refer to perpetration (e.g., ‘I punched, kicked or pushed a classmate’). The items were answered on a five-point Likert scale, ranging from 1 (no) to 5 (yes, more than once per week). In this study, questions referred to incidences occurring in the past two months. The reliability of the scale was good (victimization α = 0.75, Ω = 0.75 and perpetration α = 0.75, Ω = 0.75).

Cyberbullying was measured with the Spanish version of the European Cyberbullying Intervention Project Questionnaire (ECIP-Q) [61,62,63]. It has 22 items, divided into 11 items on cybervictimization (e.g., ‘Somebody has said offensive words to me or has insulted me using their phone or the Internet’) and 11 items on cyberperpetration (e.g., “I have isolated or ignored somebody on social media or chats”). The items were answered on a five-point Likert scale, ranging from 1 (no) to 5 (yes, more than once per week), and referred to incidences occurring in the past two months. The questionnaire had a good reliability (cybervictimization α = 0.86, Ω = 0.86 and cyberperpetration α = 0.92, Ω = 0.92).

Self-esteem was measured using Rosenberg’s Self-Esteem Scale (RSES). It was created by Rosenberg (1989) and later validated in Spain with a sample of adolescents [64]. The scale consists of10 items focused on different aspects of self-esteem (e.g., ‘In general, I am satisfied with myself’). It is answered on a four-point Likert scale, ranging from 1 (completely disagree) to 4 (completely agree), with acceptable reliability (α = 0.67, Ω = 0.68).

Empathy was measured with the Spanish version of the Basic Empathy Scale [44], adapted and reduced [51]. It has two scales: affective empathy (e.g., ‘After being with a friend who is sad about something, I usually feel sad’) and cognitive empathy (e.g., ‘When someone is feeling ‘down’, I can usually understand how s/he feels’). The first scale contains four items, and the second scale contains five items. Items are answered on a five-point Likert like scale, ranging from 1 (completely disagree) to 5 (completely agree). The scale has good reliability (α and Ω affective empathy = 0.77 and α and Ω cognitive empathy = 0.84).

Social skills were measured with the Social Skills Evaluation Scale [51], which consists of 12 items answered on a seven-point Likert scale, ranging from 1 (completely false) to 7 (completely true). Some of them are affirmative questions, while others are negative questions. Items are divided into three scales: communicative or relationship skills with five items (e.g., ‘I struggle to start a conversation with a stranger’), assertiveness with three items (e.g., ‘I usually praise and congratulate my peers when they do something right’), and conflict resolution skills with four items (e.g., ‘When two friends have an argument, they usually come to me for help’). These subscales had acceptable reliability (α and Ω communicative skills = 0.78, α and Ω assertiveness = 0.66, and α and Ω conflict resolution = 0.69).

### 2.3. Procedure

The study was conducted as a part of a cooperation for development project, including the University of Cordoba (Spain) and La Restinga Association (Peru). Within this project, Loreto’s Regional Education Department was contacted, and data were collected as a part of the current agreement between these institutions. First, a group of psychologists from La Restringa Association revised the questionnaires to find possible linguistic differences between its original version validated in Spain and expressions used in the Peruvian Amazonia. Several terms were changed (i.e., “piratear” was substituted with “hackear”, and “ordenador” was substituted with “computadora”). Then, six school directors were contacted and asked for cooperation, and four agreed to participate. Once the study was approved by each school, one class from each grade was randomly selected to fill in the survey. Parents were informed about the study and had the right to decline to participate. Participants were included if they were in any grade (1 to 6) of secondary education, had parental consent, and agreed to participate. Participants were excluded if they did not have parental consent or if they declined to participate in the study.

Participants received detailed instructions on how to fill in the questionnaires. After obtaining participants’ consent to take part in the study, data were collected anonymously and voluntarily, under the supervision of a researcher. Participants had the right to withdraw from the study at any time. No personal information that could identify the participants was collected. National and international research ethics regulations, including the Declaration of Helsinki and the Personal Data Protection Law, were followed. Procedure was approved by the Ethics Committee of the University of Cordoba, Spain (PSI2016-74871-R).

### 2.4. Data Analyses

In order to analyze the psychometric properties of the European Bullying Intervention Project Questionnaire, and the European Cyberbullying Intervention Project Questionnaire, Confirmatory Factor Analyses were conducted with the original factor structure of the questionnaires. To check if the data collected in this study fit the original factor structure, analyses were conducted with the EQS 6.2 software (Multivariate Software, Inc., Encino, CA, USA) [65]. The estimation methods were the robust maximum likelihood method and polychoric correlations, given the ordinal scales of the variables. Model fit was tested through different indices such as comparative fit index (CFI), normed-fit index (NFI), and non-normed fit index (NNFI), bigger than 0.90, and root mean square error of approximation (RMSEA) below 0.08 [66]. 

Reliability was estimated with the FACTOR 10 (Universitat Rovira i Virgili, Tarragona, Spain) [67] software. Reliability coefficients included Cronbach’s alpha and McDonald’s omega, both calculated using the polychoric correlations matrix.

Prevalence rates of involvement in different bullying and cyberbullying roles were analyzed with PASW-Statistics 20 (IBM, Armonk, NY, USA) [68]. Students were classified into different bullying and cyberbullying roles based on their responses to the items focusing on victimization and perpetration. Participants who responded never, once, or twice to all the items on perpetration and victimization were considered uninvolved. Participants who responded once or twice per month or more to any item on aggression, and responded never, once, or twice to all items on victimization, were considered bullies (and vice versa for the victims). Participants who responded once or twice per month or more to any item on both perpetration and victimization were considered bully-victims.

Descriptive analyses were performed to discover prevalence rates of different bullying and cyberbullying roles. Direct relations among variables were analyzed with Pearson’s correlations. Unique relations between predictors (self-esteem, empathy, and social skills) and different bullying and cyberbullying roles were analyzed with multinomial logistic regression analysis.

## 3. Results

**Figure 1:**
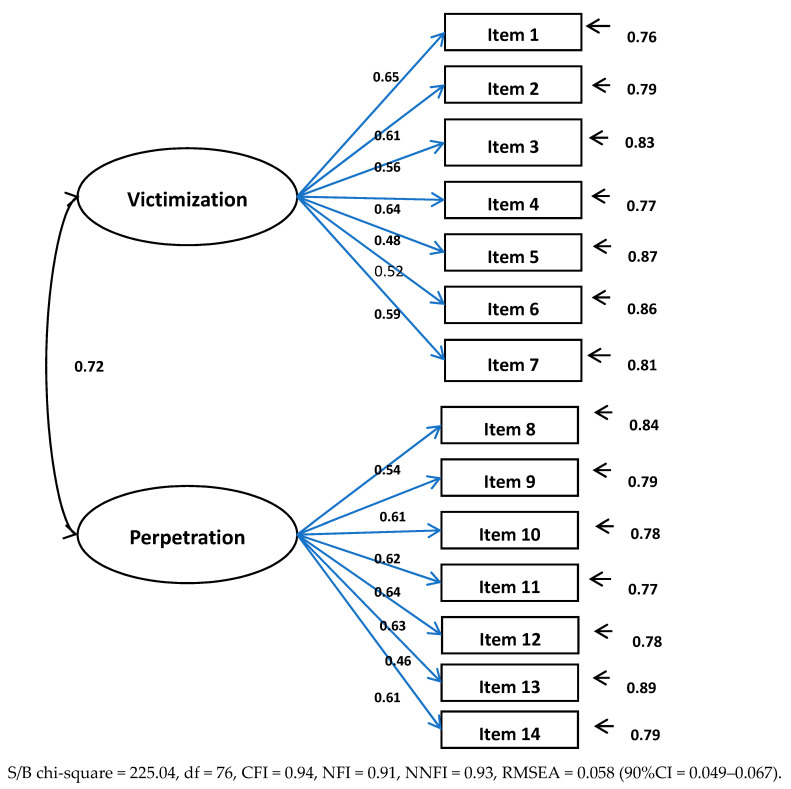
Confirmatory Factor Analyses of the European Bullying Intervention Project Questionnaire.

**Figure 2:**
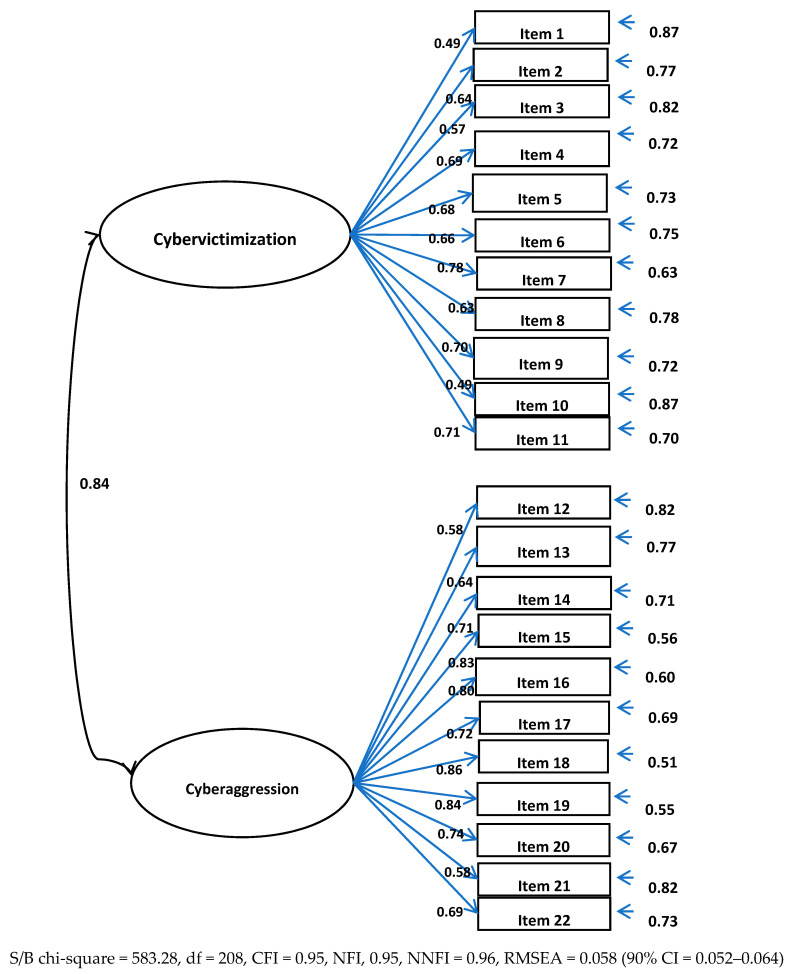
Confirmatory Factor Analyses of the European Cyberbullying Intervention Project Questionnaire.

Results of the Confirmatory Factor Analyses showed that both instruments had good psychometric properties. Factor loadings and model fit indices were adequate. Details are specified in Figure 1 and Figure 2.

**Figure 3:**
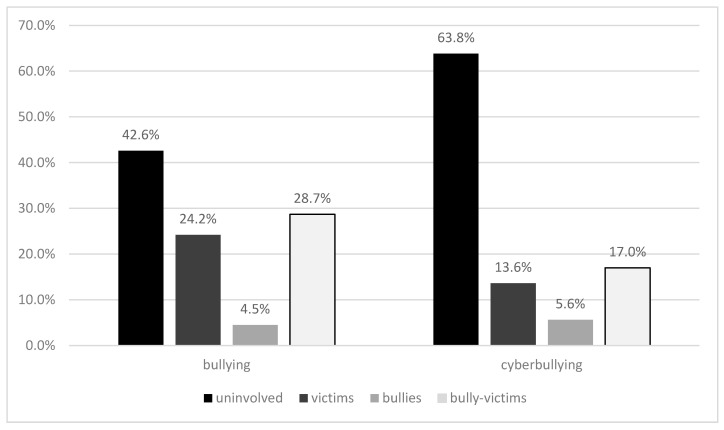
Prevalence rates of involvement in different bullying and cyberbullying roles.

The percentages of students involved in each bullying and cyberbullying role are shown in Figure 3. As can be seen in Figure 3, there are more participants involved in bullying than in cyberbullying. In both phenomena, bully-victim is the most common role.

Regarding the overlap between the bullying and cyberbullying roles, Table 1 shows that only 36.7% of students were not involved in any bullying or cyberbullying role.

Table 2 shows Pearson’s correlations among the study variables. Positive significant correlations were found between victimization and perpetration in both bullying and cyberbullying. Victimization and perpetration of cyberbullying were both related to low self-esteem. Only victimization of bullying, but not perpetration, was related to low self-esteem. Victimization (but not perpetration) of both bullying and cyberbullying were related to high affective and cognitive empathy. Bullying and cyberbullying perpetration were related to low assertiveness. There were no other significant relations between bullying, cyberbullying, and social skills.

Table 3 shows the results of the multinomial regression analysis, with bullying roles as dependent variables and self-esteem, empathy, and social skills as predictors (Nagelkerke R^2^ = 0.19). Low self-esteem and high affective empathy uniquely predicted victimization. Being a bully was uniquely related to high assertiveness. Being a bully-victim was uniquely related to being older, male, having low self-esteem, and having low assertiveness.

Table 4 shows the results of multinomial regression analysis including age, gender, self-esteem, empathy, and social skills as predictors of different cyberbullying roles (Nagelkerke R^2^ = 0.13). The variables that uniquely predicted being a cybervictim were being older and having higher cognitive empathy. Cyberbullies reported higher affective empathy. The role of the cyberbully-victim was predicted by being older, male, having low self-esteem, and having higher affective empathy.

## 4. Discussion

One of the most researched interpersonal violent phenomena occurring in childhood and adolescence are bullying and cyberbullying, as these types of violence have serious short- and long-term consequences. A literature review showed that most of the studies about bullying and cyberbullying have been conducted in wealthy, Western countries [1]. Nevertheless, knowledge about violence, including associated risk and protective factors, is needed the most in disadvantaged areas, including many regions of Latin America [2,3,13,14,15]. This knowledge can only be gathered using validated measurement tools. The current study uncovered good psychometric properties of the European questionnaires EIBP-Q and ECIP-Q [61,62,63], when used to measure bullying and cyberbullying in Peru. These instruments were also found to have good psychometric properties for other Latin American countries, such as Colombia [16,17] and Ecuador [3]. Thus, bullying and cyberbullying seem to have similar characteristics and factor structures in different geographical areas, including certain understudied regions, such as rainforests.

Scientific evidence from this study showed that both the EBIP-Q and ECIP-Q can be used to measure bullying and cyberbullying in the Peruvian Amazonia. This region is one of the most economically depressed and educationally disadvantaged in Peru. Thus, both questionnaires can be very useful to detect and help eradicate bullying and cyberbullying among Peruvian adolescents. Having these instruments available also broadens horizons for research focused on bullying and cyberbullying in Peru. Based on the current results, it will also be possible to conduct cross-national and transcultural comparative studies in different countries, including Peru, Colombia, and Ecuador, as well as other more distant European countries.

There is a wide variety of ways to define and measure bullying and cyberbullying [61,62]. For this reason, comparing their prevalence rates across different countries is still a scientific challenge. The results of this study—obtained based on definitions, methods, and instruments that had been broadly used and validated in other countries—contribute to the advancement of this research line. 

This study showed that 4.5% of students reported bullying perpetration, 24.2% reported being victims, and 28.7% reported being bully-victims. As a whole, the percentage of victims was similar to the one found by Fleming and Jacobsen [18] in 19 countries around the world. However, the prevalence rate of students involved as bully/victims was high in comparison to many other countries [1,19]. Thus, the current study showed that, as also pointed out by previous research, school violence in some Latin American countries is higher than in European or North American countries [22]. With respect to Latin American countries, this study showed a higher prevalence rate of bullying in the Peruvian Amazonia, in comparison to the average estimated rates for Peru [15]. The results of this study showed that the prevalence rate of bullying involvement is mostly similar to those reported through surveys conducted by Peruvian government [25,26,27,28]. Given that this study reported results obtained with validated and reliable instruments, it is possible to conclude, from a transcultural and intracultural comparison, that bullying is a serious problem in the Peruvian Amazonia.

This study also found that 5.6% of participants reported cyberperpetration, 13.6% reported cybervictimization, and 17% reported being cyberbully-victims. This means that one out of three participants was involved in cyberbullying, which is more than the average cross-national prevalence rate of around one out of five [1,19]. When compared to geographically close countries, involvement rates are within the expected range, although they might be considered to be medium-high [15,24]. When compared to the few existent studies in Peru [30,31], involvement in cyberbullying victimization was slightly lower in the current study. Still, in contrast to other regions of Peru, the Peruvian Amazonia is a particularly economically disadvantaged area with reduced Internet access and limited access to digital devices and smartphones. Therefore, the prevalence rates of cyberbullying are particularly disturbing and prevention, and intervention programs are urgently needed.

There is a noteworthy overlap between bullying and cyberbullying. According to this study, one out of three students is involved in both problem behaviors. This finding falls in line with the results of other studies that showed that bullying and cyberbullying tend to form patterns of antisocial behaviors [12]. There is a relationship between victimization in face-to-face and virtual contexts. Therefore, bullying and cyberbullying prevention and intervention programs in Peru should focus on both interconnected problem behaviors. These comprehensive programs should include components related to both face-to-face behavior and cyberbehavior.

In accordance with previous scientific literature reviews, it was expected that victimization and perpetration of bullying [38,41] and cyberbullying [39,40] would be negatively related to self-esteem. The results of the current study confirmed these expectations, except when it came to the relationship between bullying perpetration and self-esteem. This finding differs from the conclusions of most studies around the world [36], possibly indicating a distinctive feature of adolescents in the Peruvian Amazonia. These results should be confirmed in future studies.

In the current study, bullying and cyberbullying victimization were found to be positively related to both affective and cognitive empathy. However, no relationship was found between bullying perpetration, and affective and cognitive empathy. This seems to reinforce certain researchers’ theory that victims are more sensitive than others [49], since adolescents with high empathy may be able to anticipate the emotional impact that aggression has on the victim and, thus, be dissuaded from hurting others [48]. On the other hand, these findings differ from some hypotheses that present bullies as highly empathetic but, at the same time, manipulative and Machiavellian [50].

Social skills such as conflict resolution and communication were not related to bullying and cyberbullying. The results obtained in the current study, with adolescents from the Peruvian Amazonia, are not consistent with what has been observed in other studies conducted in different places around the world [40,56,57]. Only assertiveness was negatively related to bullying and cyberbullying perpetration, which is a similar finding to some previous studies [58]. It is possible that these social skills are not applicable in real-life situations. Therefore, specific training in social competence, and not only social skills, could be beneficial.

It was also found that low self-esteem and high affective empathy were predictors of victimization in adolescents from the Peruvian Amazonia. This is consistent with the findings of a study conducted recently in its neighboring country, Ecuador, with the same methods and instruments [3]. These findings reinforce previous findings from studies in other countries that showed high levels of empathy in victims of bullying [37,46].

Being a bullying perpetrator was predicted by high assertiveness. This contradicts other findings that showed that low social skills predicted more peer aggression [59]. It is also inconsistent with the findings of a study conducted in Ecuador [3] that showed that aggression was predicted by low self-esteem and low cognitive empathy. None of the tested social skills (communication, assertiveness, and conflict resolution) showed predictive value. Moreover, being a bully-victim was predicted by being older, male, having low self-esteem, and having low assertiveness.

Being a cybervictim was predicted by being older and having higher levels of cognitive empathy. The predictive role of empathy with respect to cybervictimization has been described previously in some studies [32], although most of the studies found high levels of affective empathy in victims [42]. There were also several research studies that did not find a significant relationship between empathy, bullying, and cyberbullying [37,40]. Being a cyberbully was predicted by a higher level of affective empathy. Being a cyberbully-victim was predicted by being older, male, having low self-esteem, and having higher levels of affective empathy. Thus, high affective empathy predicted both being a cyberbully and a cyberbully/victim, which is a rather unexpected finding. This could indicate that cyberaggression, unlike physical aggression, is associated with a profile of people who use their high affective empathy to harm and subdue their peers [50]. Future studies should be conducted to explain these results.

## 5. Conclusions

The current study provides further evidence for the predictive role of self-esteem, empathy, and social skills, in relation to bullying and cyberbullying. Thus, preventing and reducing bullying and cyberbullying in Peruvian adolescents requires designing and implementing education strategies that enhance self-esteem, empathy, and social skills. These strategies must be implemented throughout the school years, starting in early education, in order to boost prevention efforts. This is important, because it was found that certain bullying and cyberbullying roles were predicted by being older. Gender should also be taken into account when developing strategies, as being male is also a predictor of bullying and cyberbullying. Moreover, programs should focus on applying self-esteem, empathy, and social skills to real-life situations, not only to help students improve their social skills but also to help them develop competencies for behaving in a prosocial way.

This study had several limitations. Certain limitations were related to the research methods, namely, that the study was cross-sectional and conducted through self-reports. Although predictions were made on a theoretical basis, future longitudinal studies should confirm the results of this study while disentangling predictors from correlates and consequences. The use of peer-reports, combined with self-reports, could contribute to higher accuracy of results in future studies. The low reliability registered after the use of Rosenberg’s self-esteem scale also limits the interpretation of the current findings.

More research on bullying and cyberbullying is needed, especially in more vulnerable countries with less economic development. Comparative studies involving both wealthier participants and participants from disadvantaged geographical areas could be especially interesting. Using validated instruments, such as those used in the current study, is key to allowing transcultural comparisons. Scientific evidence is particularly valuable when making adequate political decisions in education, such as designing and implementing bullying and cyberbullying prevention and intervention programs that are adapted to cultural characteristics and needs.

## Figures and Tables

**Table 1:** Prevalence rates taking into account the overlap among bullying and cyberbullying roles.

Roles	Uninvolved in Cyberbullying	Cybervictim	Cyberbully	Cyberbully/Victim
Uninvolved in bullying	36.7%	3.1%	1.2%	1.7%
Victim	15.5%	5.2%	0.8%	3.3%
Bully	2.3%	1%	0.8%	0.4%
Bully-victim	9.9%	4.3%	2.5%	11.3%

Chi-square _(9)_ = 133.17, *p* < 0.01, V = 0.28, *p* < 0.01.

**Table 2:** Correlations among the study variables.

Study Variables	1	2	3	4	5	6	7	8	9
1. Victimization	1								
2. Bullying perpetration	398 **	1							
3. Cybervictimization	0.468 **	0.323 **	1						
4. Cyberperpetration	0.347 **	0.471 **	0.603 **	1					
5. Self-esteem	−215 **	−0.083	−0.152**	−0.111 *	1				
6. Affective empathy	0.205 **	0.012	0.165 **	0.071	0.005	1			
7. Cognitive empathy	0.133 **	0.008	0.091 *	−0.021	0.163 **	0.591 **	1		
8. Communicative skills	0.068	−0.016	−0.004	−0.004	−0.092 *	0.248 **	0.192 **	1	
9. Assertiveness	−0.041	−0.111**	0.004	−0.117 **	0.217 **	0.348 **	0.437 **	0.307 **	1
10. Conflict resolution	−0.001	−0.057	0.038	−0.049	0.171 **	0.389 **	0.395 **	0.270 **	0.612 **

* *p* < 0.05, ** *p* < 0.01.

**Table 3:** Multinomial regression with age, gender, self-esteem, empathy, and social skills as predictors of different bullying roles.

Predictors	Victims	Bullies	Bully-Victims
	OR (95% CI)	OR (95% CI)	OR (95% CI)
Age	1.04 (0.89−1.21)	0.98 (0.74−1.30)	1.22 (1.06−1.41)
Male	1.21 (0.71−2.08)	2.80 (0.94−8.36)	2.60 (1.54−4.38)
Self-esteem	0.91 (0.85−0.97)	1.03 (0.90−1.18)	0.91 (0.86v0.97)
Affective empathy	1.10 (1.01−1.19)	1.10 (0.94−1.29)	1.03 (0.96−1.11)
Cognitive empathy	1.06 (0.98−1.15)	0.94 (0.82−1.08)	1.05 (0.98−1.12)
Communicative skills	1.03 (0.99−1.07)	1 (0.93−1.06)	0.99 (0.96−1.02)
Assertiveness	0.93 (0.86−1)	1.25 (1.04−1.50)	0.90 (0.84−0.97)
Conflict resolution	0.96 (0.90−1.02)	0.92 (0.82−1.02)	1.02 (0.96−1.08)

Note: All comparisons were made using the uninvolved as a reference category.

**Table 4:** Multinomial regression with age, gender, self-esteem, empathy, and social skills as predictors of different cyberbullying roles.

Predictors	Cybervictims	Cyberbullies	Cyberbully-Victims
	OR (95% CI)	OR (95% CI)	OR (95% CI)
Age	1.27 (1.06−1.52)	1.25 (0.96−1.63)	1.19 (1.01−1.40)
Male	0.79 (0.41−1.49)	2.12 (0.76−5.91)	1.85 (1.01−3.39)
Self-esteem	0.97 (0.89−1.04)	0.96 (0.85−1.08)	0.93 (0.86−.99)
Affective empathy	0.92 (0.84−1.01)	1.22 (1.04−1.44)	1.11 (1.01−1.22)
Cognitive empathy	1.11 (1.02−1.21)	0.95 (0.83−1.09)	1 (0.92−1.09)
Social skills	0.99 (0.95−1.03)	0.98 (0.92−1.05)	0.99 (0.95−1.03)
Assertiveness	1.04 (0.95−1.15)	0.93 (0.81−1.07)	0.94 (0.86−1.02)
Conflict resolution	0.98 (0.91−1.05)	0.94 (0.84−1.05)	1.02 (0.95−1.09)

Note: All comparisons were made using the uninvolved group as a reference category.
